# Benefits of musculoskeletal health promotion in school communities through service-learning: a mixed-method approach

**DOI:** 10.3389/fpubh.2025.1507730

**Published:** 2025-03-06

**Authors:** Sandra Calvo, Rocío Fortún-Rabadán, Sara Pérez-Palomares, Beatriz Carpallo-Porcar, Raquel Lafuente-Ureta, Carolina Jiménez-Sánchez

**Affiliations:** ^1^Faculty of Health Sciences, Department of Physiatry and Nursing, University of Zaragoza, Zaragoza, Spain; ^2^Aragón Health Research Institute, Zaragoza, Spain; ^3^Faculty of Health Sciences, Department of Physical Therapy, Universidad San Jorge, Villanueva de Gállego, Spain

**Keywords:** health promotion, health education, school community, physical therapy, service-learning, mixed-methods

## Abstract

**Background:**

Service-learning (S-L) is an educational method that enhances social and civic engagement among health science students, addressing unmet community needs. Musculoskeletal disorders (MSKD) represent a public health issue with increasing prevalence among school-aged population as reported by at least 1 on 5 children. Prevention programs for MSKD in schools are scarce, although evidence supports the efficacy of health education provided by physical therapists. This study aimed to evaluate the impact of a musculoskeletal health promotion program through S-L on school students, teachers, and physical therapy students.

**Methods:**

A mixed-methods approach was used. A quasi-experimental design assessed the learning and satisfaction of school students and teachers using pre-and post-intervention questionnaires. Additionally, qualitative data from reflective diaries of fourth-year physical therapy students were analyzed to capture their experiences. The intervention was conducted in 10 schools in Spain over two academic years, targeting children aged 10–14 and their teachers.

**Results:**

A total of 1,051 school students and 32 teachers participated. Pre-post evaluations revealed significant learnings on MSKD prevention and body awareness in both groups (*p* < 0.05), highlighting the increase in knowledge about MSKD prevention and self-treatment for school students and on MSKD derived from inadequate postures for schoolteachers. Satisfaction was high, with students and teachers rating the program 4.4/5. Teachers highlighted the value of training for their professional practice, while students appreciated learning about self-care. Physical therapy students were 4 and reflected on their motivation for the project, learning experiences, as well as positive and negative aspects of the service.

**Discussion:**

The S-L community-based intervention effectively increased awareness of musculoskeletal health and self-management skills. The peer learning environment and participatory approach encouraged engagement and knowledge retention in both students and teachers.

**Conclusion:**

S-L is a valuable strategy for promoting musculoskeletal health in schools while improving health education skills among the future physical therapists through experiential learning.

## Introduction

Service-learning (S-L) is a valuable resource to promote public health education and social engagement among students in health sciences, as essential parts in the curriculum of future healthcare professionals (1, 2). Cooperation and civic competencies are conveyed through this learning methodology, whose principles are based on critical reflection, mutual benefit, and experiential learning (3, 4), while the service improves the health of the communities (5, 6). This service is particularly useful for covering disregarded needs in specific collectives, that can benefit from health initiatives with no economic cost (7, 8). Among others, S-L programs focused on health education have demonstrated their usefulness when developed within the school community (4, 9).

Musculoskeletal disorders (MSKD) represent a public health problem, as leading cause of chronic pain and disability worldwide (10, 11). During the last decades, MSKD are affecting a growing number of people and occurring at younger age (12, 13), with prevalence data ranging from 20 to 40% when assessing chronic musculoskeletal and back pain among school-aged children (14). Furthermore, children reporting musculoskeletal pain are also more likely to suffer it during adolescence and young adulthood (15). Different factors appear to be related to the increasing prevalence in the early life stages, such as lower socioeconomic status (15), psychological distress (16), or a sedentary lifestyle (17), which is associated with obesity (18) and poor ergonomics (17).

Despite the burden of musculoskeletal conditions, health policies are not facing this problem satisfactorily (19, 20). The lack of awareness and investment in both research and preventive interventions is pointed. Whereas educational strategies conducted by physical therapists show to boost the effectiveness of rehabilitation in people with MSKD (21), these key interventions are not generally offered with preventive aims at the primary care level (19). Concretely, the school population is especially overlooked (22), and this scenario is even more disturbing since the diagnosis and management of pain, once it has become chronic, show uncertain efficacy in children (23).

Increasing knowledge in terms of body awareness (24, 25), healthy habits (22, 26), and self-management of pain or injuries (27, 28), is helpful to preventing the development of MSKD (28). Indeed, confidence in self-care strategies, achieved through increasing health literacy, is a meaningful resource for people presenting chronic musculoskeletal pain (19, 29). There is still no data from cohorts of children followed long-term after musculoskeletal health promotion programs (MSKHPP), which reinforces the dismissed needs in terms of prevention. However, it can be assumed that such training during the early stages of life can promote useful, lifelong learning for the primordial prevention of MSKD. Physical therapists are in an ideal position to address these aspects of education (29, 30), filling the current gaps in musculoskeletal health promotion among children and adolescents (12, 22).

Additionally, the benefits of health education interventions could be enhanced when conducted at school, driven by facilitatory aspects such as the peer context (31, 32). The training received by teachers, allowing them to implement this knowledge in the day-to-day school activities, as well as the fact that pupils and teachers learn together in a non-hierarchical relationship (33, 34), could also positively contribute. In addition, the cooperation values conveyed by S-L can promote motivation and learning in both student collectives (4, 35). However, despite these benefits can be hypothesized, the usefulness of S-L programs conducted for school-age children remains understudied. Finally, in addition to quantitative analysis to assess the interventions, qualitative approaches are important to enrich research on S-L programs, allowing to target specific aspects, and even capturing new beliefs, needs and barriers (4).

This study aimed to use quantitative and qualitative approaches to assess the learning and satisfaction of school students and teachers after participating in a MSKHPP conducted at school, as well as to assess fourth-year physical therapy students´ perceptions of their experiences in this S-L project.

## Materials and methods

### Study design

A concurrent triangulation mixed method design was selected for this study. It was conducted in accordance with the Declaration of Helsinki and following the guidelines of the Mixed Method Article Report Standards (MMARS) (36).

A quantitative approach with a quasi-experimental pre-post design was carried out to assess the learning by the school community. Quantitative data on satisfaction with the MSKHPP received was also collected from the school students and teachers.

A qualitative approach was performed to evaluate the experiences of the physical therapy students providing the program. Their reflective diaries were collected and examined by means of a content analysis. This project was conducted as part of the Physical Therapy Degree S-L Program, which includes various community interventions, and was approved by the Ethics Committee of Universidad San Jorge (008–18/19).

### Setting and sample

The MSKHPP was carried out in different public and state-funded schools in Zaragoza in two consecutive academic courses, 2017–18 and 2018–19, thanks to an educational campaign addressing health and disability issues for school community. Inclusion criteria for school participants were: (1) children aged between 10 and 14 years (from the fifth grade of primary school to the first year of secondary school) and their teachers, and (2) signing the informed consent form. The exclusion criterion was the refusal to participate in the study.

Participants providing the service were students in the 4th year of the Physical Therapy Degree at the Universidad San Jorge, who developed their final degree project during 2017–18 or 2018–19 academic years. The students were supervised by the same academic tutors, which ensured the repeatability of the service. The sample was selected following a purpose recruitment, through an email request sent by the researchers. The inclusion criteria were as follows: (1) being 18 years or more, being a student of 4th course in Physical Therapy Degree at University San Jorge, (2) being enrolled in the Final Degree Project subject, (3) carrying out a S-L Project as theme of work for their final degree project, and (4) signing the informed consent form. The exclusion criterion was the refusal to participate in the study.

### Intervention

The present S-L intervention consisted of a theoretical and practical musculoskeletal health education workshop led by fourth-year physical therapy students. The average duration was 60 min and involved both school students and teachers´ participation simultaneously. The program was designed to address the prevention of the main factors associated to the development of MSKD in children and adolescents. Additionally, the S-L principles (37) were considered, i.e., to have a social (in the school community) and pedagogical impact (on university students).

The theoretical session was planned to identify and address the gaps in the knowledge about musculoskeletal health in the school community. For this purpose, a participatory talk was carried out tackling anatomic-physiological knowledge of the musculoskeletal system, concepts of body awareness, prevention and self-management of MSKD and the importance of healthy habits such as physical activity or sleep routines.

Guided practical activities were then conducted. Flexibility through stretching exercises (38), safe strengthening exercises (38), and self-massage techniques (39), including sustained inhibitory pressure, were practiced for the main muscle groups of the lower limb, upper limb, neck, thoracic, and low back spine. These were accompanied by the practice of deep-breathing techniques (40) and interspersed with active education on the ergonomics and timing of the sitting position, and backpack carrying (41). All the contents were first demonstrated by the physical therapy students and then practiced by children while guiding their attention to their bodies, with the aim of improving proprioception (42). This allowed the recognition of musculoskeletal sensations such as contraction, relaxation, elongation, spinal alignment, muscle contracture, and pain threshold, concepts that were introduced during the previous theoretical intervention.

The teaching methods were selected to stimulate students´ motivation and learning. Role-playing, guided discovery, and participation were particularly encouraged (43). The session concluded by highlighting the key aspects to be reinforced by teachers on the courses.

On the other hand, to cover the pedagogical impact of the S-L methodology, several aspects were considered. First, the sessions were proposed in a symmetrical situation in which the physical therapy students led the sessions and at the same time participated as an integrated element belonging to the school community. This was intended to enhance the cooperation between both students’ collectives, fostering motivation and active learning as key parts of S-L. In addition, the engagement of the physical therapy students in health promotion was encouraged through the development of their final degree project, by feeling the service as their own project. The intervention was also designed to improve essential skills for health promotion among the future physical therapists, such as applying conceptual (scientific) content in a real-life context and developing communication skills. Moreover, embedding all these aspects in a solidarity purpose make learning strongly meaningful.

### Assessment

#### Quantitative data collection

Socio-demographic data such as gender, age, role and, only in case of schoolteachers, the number of years they have worked in education, was collected through a paper-based table. The efficacy of the MSKHPP was evaluated using a pre-and post-test design to determine the increase in knowledge in the school communities. For this purpose, a self-administered, anonymous *ad hoc* questionnaire was designed, including 5 Likert-scale questions from 1 to 5 (1: strongly disagree; 2: disagree; 3: neutral; 4: agree; 5: strongly agree) related to the content of the program (Table 1). The questionnaire was completed by the participants immediately before (pre-test) and after (post-test) the intervention, in two different paper-based copies. The increase in knowledge was calculated by comparing the post and pre-test values for each domain assessed. In addition, at the end of the intervention, the satisfaction and usefulness of the program were evaluated using a self-administered anonymous questionnaire with five items on a 5-Likert-scale (1: strongly disagree; 2: disagree; 3: neutral; 4: agree; 5: strongly agree) (Table 2). An open-ended question was included to highlight the most relevant aspects of the intervention.

**Table 1:** *Ad-hoc* pre-post intervention questionnaire to assess the musculoskeletal health education workshop.

Questions	Population	Pre-intervention						Post-intervention						*p*-value
		*N* (%)					Mean ± SD Median (IQR)	*N* (%)					Mean ± SD Median (IQR)	
		Strongly disagree (1)	Disagree (2)	Neutral (3)	Agree (4)	Strongly agree (5)		Strongly disagree (1)	Disagree (2)	Neutral (3)	Agree (4)	Strongly agree (5)		
1. Knowledge about the locomotor system	School students	104 (9.9)	132 (12.6)	326 (31)	337 (32.1)	152 (14.5)	3.25 ± 1.20 3 (1)	18 (1.7)	50 (4.8)	144 (13.6)	338 (32.1)	501 (47.7)	4.19 ± 0.96 4 (1)	<0.001**^W^
	School teachers	0 (0)	4 (12.5)	7 (21.9)	14 (43.8)	7 (21.8)	3.75 ± 0.95 4 (3)	0 (0)	2 (6.3)	4 (12.5)	17 (53.1)	9 (28.1)	4.03 ± 0.82 4 (3)	0.004*^W^
2. Knowledge about MSKD	School students	130 (12.4)	246 (23.5)	219 (20.8)	271 (25.8)	185 (17.6)	3.11 ± 1.25 3 (2)	24 (2.3)	60 (5.7)	152 (14.5)	442 (42)	373 (35.5)	4.02 ± 0.96 4 (1)	<0.001**^W^
	School teachers	0 (0)	0 (0)	4 (12.5)	19 (59.4)	9 (28.1)	4.16 ± 0.63 4 (2)	0 (0)	0 (0)	2 (6.3)	6 (18.8)	24 (75)	4.68 ± 0.59 5 (2)	<0.001**^W^
3. Knowledge about physical therapy	School students	165 (15.7)	167 (15.9)	247 (23.5)	267 (25.4)	205 (19.5)	3.33 ± 1.26 3 (2)	21 (2)	57 (5.4)	191 (18.2)	343 (32.6)	428 (40.7)	4.05 ± 0.99 4 (2)	<0.001**^W^
	School teachers	0 (0)	1 (3.2)	1 (3.2)	18 (59.4)	12 (31.3)	4.27 ± 0.68 4 (3)	0 (0)	0 (0)	1 (3.2)	4 (12.5)	27 (84.3)	4.82 ± 0.47 5 (2)	<0.001**^W^
4. Knowledge about MSKD prevention and self-treatment	School students	130 (12.4)	125 (11.9)	216 (20.5)	284 (27)	296 (28.1)	3.26 ± 1.29 3 (2)	39 (3.7)	72 (6.8)	167 (15.9)	406 (38.6)	367 (34.9)	4.32 ± 0.89 5 (1)	<0.001**^W^
	School teachers	1 (3.2)	1 (3.2)	2 (6.3)	15 (46.8)	13 (40.5)	4.18 ± 0.93 4 (4)	0 (0)	0 (0)	2 (6.3)	4 (12.5)	26 (81.2)	4.75 ± 0.57 5 (2)	0.004*^W^
5. Self-body awareness in different situations.	School students	139 (13.2)	159 (15.1)	269 (25.6)	249 (23.7)	235 (22.4)	3.72 ± 1.11 4 (2)	15 (1.4)	27 (2.6)	135 (12.8)	298 (28.4)	576 (54.8)	4.24 ± 0.81 4 (1)	<0.001**^W^
	School teachers	1 (3.2)	1 (3.2)	2 (6.3)	16 (50)	12 (37.4)	4.13 ± 0.91 4 (2)	0 (0)	1 (3.2)	2 (6.3)	11 (34.4)	18 (56.2)	4.44 ± 0.76 5 (2)	0.002*^W^
6. Knowledge about MSKD derived from inadequate postures.	School students	55 (5.2)	121 (11.5)	260 (24.7)	328 (31.2)	287 (27.3)	3.59 ± 1.25 4 (2)	3 (0.3)	30 (2.8)	143 (13.6)	406 (38.6)	469 (44.6)	4.30 ± 0.90 5 (1)	<0.001**^S^
	School teachers	2 (6.3)	4 (12.5)	7 (21.9)	14 (43.8)	5 (15.6)	3.47 ± 1.07 4 (5)	0 (0)	1 (3.2)	2 (6.3)	8 (25)	21 (65.6)	4.54 ± 0.76 5 (3)	<0.001**^W^
7. Usefulness of knowledge on MSKD prevention.	School students	102 (9.7)	135 (12.8)	256 (24.4)	293 (27.9)	265 (25.2)	3.44 ± 1.22 4 (1)	21 (2)	54 (5.1)	209 (19.9)	412 (39.2)	355 (33.8)	4.22 ± 0.97 5 (1)	<0.001**^W^
	School teachers	0 (0)	2 (6.3)	2 (6.3)	19 (59.4)	9 (28.1)	4.08 ± 0.78 4 (4)	0 (0)	0 (0)	1 (3.2)	9 (28.1)	22 (68.8)	4.54 ± 0.76 5 (3)	0.002*^W^
8. Interest in MSKD prevention activities.	School students	82 (7.8)	132 (12.6)	209 (19.9)	322 (30.6)	306 (29.1)	3.60 ± 1.17 4 (2)	15 (1.4)	33 (3.1)	132 (12.5)	313 (29.8)	558 (53.1)	4.41 ± 0.91 5 (1)	<0.001**^W^
	School teachers	0 (0)	0 (0)	3 (9.4)	16 (50)	13 (40.6)	4.31 ± 0.65 4 (2)	0 (0)	0 (0)	2 (6.3)	12 (37.5)	18 (56.2)	4.50 ± 0.62 5 (2)	0.031*^W^
9. Curiosity about MSKD prevention.	School students	93 (8.8)	148 (14.1)	271 (25.8)	280 (26.7)	259 (24.6)	3.59 ± 1.25 4 (2)	18 (1.7)	51 (4.8)	146 (13.9)	296 (28.1)	540 (51.4)	3.94 ± 1.05 4 (2)	<0.001**^W^
	School teachers	0 (0)	1 (3.2)	1 (3.2)	11 (34.3)	19 (59.4)	4.50 ± 0.72 5 (3)	0 (0)	0 (0)	1 (3.2)	6 (18.8)	25 (78.1)	4.75 ± 0.51 5 (2)	0.008*^W^
10. Disposal to advise others about the acquired knowledge	School students	68 (6.5)	141 (13.4)	243 (23.1)	299 (28.4)	300 (28.6)	3.55 ± 1.19 4 (2)	18 (1.7)	29 (2.8)	117 (11.1)	224 (21.3)	663 (63.1)	3.97 ± 0.96 4 (2)	<0.001**^S^
	School teachers	1 (3.2)	5 (15.6)	7 (21.9)	15 (46.9)	4 (12.5)	3.50 ± 1.02 4 (4)	0 (0)	2 (6.3)	5 (15.6)	16 (50)	9 (28.1)	4.00 ± 0.80 4 (3)	<0.001**^W^

MSKD, musculoskeletal disorders; SD, standard deviation; IQR, interquartile range.

*p < 0.05; **p < 0.001.^S^Using paired Student’s *t*-test; ^W^Using Wilcoxon signed-rank test.

**Table 2:** *Ad-hoc* program satisfaction questionnaire.

Questions	Population	*N* (%)					Mean ± SD Median(IQR)
		Strongly disagree (1)	Disagree (2)	Neutral (3)	Agree (4)	Strongly agree (5)	
Question 1. I have increased my knowledge about the human body and MSKD prevention	School students	13 (1.2)	19 (1.8)	89 (8.5)	266 (25.3)	664 (63.2)	4.48 ± 0.82 5 (1)
	School teachers	(0)	(0)	(0)	13 (40.6)	19 (59.4)	4.60 ± 0.52 5 (1)
Question 2. I have enjoyed getting to know my body	School students	15 (1.4)	23 (2.3)	116 (11.0)	307 (29.2)	590 (56.1)	4.36 ± 0.87 5 (1)
	School teachers	(0)	(0)	(0)	13 (40.6)	19 (59.4)	4.60 ± 0.52 5 (1)
Question 3. What I have learned is useful to me	School students	19 (1.8)	26 (2.5)	89 (8.5)	303 (28.8)	614 (58.4)	4.40 ± 0.88 5 (1)
	School teachers	(0)	4 (12.5)	(0)	13 (40.6)	15 (46.9)	4.20 ± 0.92 4 (3)
Question 4. What I have learned is useful to help others	School students	12 (1.1)	29 (2.8)	82 (7.8)	312 (29.7)	616 (58.5)	4.42 ± 0.84 5 (1)
	School teachers	(0)	3 (9.4)	7 (21.9)	12 (37.5)	10 (31.2)	3.90 ± 0.99 4 (3)
Question 5. I would like to participate in this type of initiatives in the future	School students	32 (3.0)	26 (2.5)	104 (9.9)	254 (24.2)	634 (60.3)	4.37 ± 0.97 5 (1)
	School teachers	(0)	(0)	(0)	10 (31.2)	22 (68.8)	4.70 ± 0.48 5 (1)

MSKD, musculoskeletal disorders; SD, standard deviation; IQR, interquartile range.

#### Qualitative data collection

To assess the experiences of physical therapy students during the program, qualitative data was collected through a reflective journal, composed of questions related to the service provided: selection process, development, needs identified, and future perspectives. The questions were open-ended but formulated in order that the physical therapy students could go deeper in different levels of reflection related to their learning experience. They filled out the diary in a text document, during the service and after it finished. Prior to the intervention, researchers performed a previous positioning procedure or “bracketing” to avoid influencing the data collection and analysis with their previous knowledge of the topic.

### Data analysis

Quantitative statistical analysis was carried out with the SPSS 28.0 version (IBM Corporation, Armonk, NY, USA). For the descriptive analysis, the mean and standard deviation (SD) or the median and interquartile range (IQR) and numbers (percentages) were used. To determine the normality of the quantitative variables, the Shapiro–Wilk test was used. The paired Student’s t-test or the Wilcoxon signed-rank test was used to compare the repeated measurements on each population. Statistical analysis was carried out at a confidence level of 95% and a statistical significance of *p* < 0.05 for all comparisons.

A qualitative descriptive approach was chosen to describe the experience of physical therapy students during the program. The written documents were analyzed using conventional content analysis (44). This methodology consists of the coding categories that emerge from the raw data through constant examination and comparison. Data was transferred to Atlas-ti (45), and read repeatedly. A further reading, word by word, was performed to establish and capture key concepts. The codes were then organized and grouped by meaning into subcategories. After, the subcategories were combined into a shorter number of categories. Finally, researchers developed definitions for each category and subcategory. Every category and subcategory were supported by appropriate quotes.

To improve trustworthiness and quality of the analysis, data triangulation was performed. Two researchers (CJ and RL) discussed and compared codes, subcategories, and categories, redefining and modifying them to reach a common agreement. Then, findings were sent to the rest of the members of the research team to be compared and discussed to get a consensus about the results.

## Results

### School community

Ten schools from Zaragoza (Spain) took part in the program. Socio-demographic characteristics of all participants are presented in Table 3.

**Table 3:** Socio-demographic characteristics.

		2017–2018		2018–2019	
		School students	School teachers	School students	School teachers
Gender	Male	288 (50.3%)	9 (45.0%)	241 (50.3%)	3 (25.0%)
	Female	284 (49.7%)	11 (55.0%)	238 (49.7%)	9 (75.0%)
Age	Male	11.1 ± 2.0	38.0 ± 9.9	12.1 ± 2.8	50.3 ± 7.0
	Female	10.9 ± 2.2	37.7 ± 5.0	11.9 ± 1.8	41.5 ± 9.8

A total of 572 school students received the intervention during the 2017–2018 school year, with an average age of 11.02 ± 2.8 years. Another 479 school students participated during 2018–2019, with their mean age being 12 ± 2.4 years. Among all school students, 50.3% were male, 49.7% female, and 38.4% of them were in the fifth grade of primary school, 40.7% in the sixth, and 20.9% in the first year of secondary school.

Regarding schoolteachers, 20 academic tutors participated in the program during the 2017–2018 academic course, with a mean age of 37.8 ± 7.9 years, followed by another 12 teachers during 2018–19, aged 44.6 ± 8.6 years on average. The majority of schoolteachers were female (62.5%, as opposed to 37.5% of males), and they had been working in education for an average of 16.6 ± 9.7 years.

Table 1 shows the results achieved in both groups during the two academic courses after the pre-and post-questionnaire analysis. In the initial assessment, children gave the lowest score to their knowledge of MSKD, and the highest score was for self-body awareness. Teachers rated their initial knowledge about MSKD derived from inadequate postures as the worst item, while their curiosity to learn about MSKD prevention scored the highest in this group.

The pre-post evaluation revealed an increase in knowledge in all the aspects assessed. The item with the greatest change was prevention and self-treatment (1.06) for the children and body awareness and MSKD derived from inadequate posture (1.07) for teachers. Specifically, significant increases in knowledge about the musculoskeletal system, MSKD, physical therapy, and prevention (questions 1–4) were revealed in both groups (*p* < 0.05). Moreover, there were significant changes in terms of self-body awareness, posture and the usefulness of the information provided (questions 5–7) (*p* < 0.05). An increase in interest, learning and education about MSKD prevention was also found (questions 8–10) (*p* < 0.05). In contrast, the item that improved the least was curiosity about MSKD prevention (0.35) among children and interest in MSKD prevention activities (0.19) among teachers. This suggests that, despite the general increase in knowledge, the children’s curiosity about MSKD prevention and the teachers’ enthusiasm for prevention were rather modest.

The results derived from the satisfaction assessment are shown in Table 2. The school students rated the MSKHPP with a total mean score of 4.40 ± 0.88/5, and teachers with a mean score of 4.40 ± 0.76/5. For school students, the increase of their knowledge about the human body and MSKD prevention was the most highly valued. In contrast, participation in this type of initiative was most important for teachers in the future. The open-ended question allowed us to extract the most frequently highlighted aspects of program evaluation. On this point, school students emphasized the increase in knowledge and the usefulness of the learning regarding self-treatment. As for the group of teachers, they valued above all their own learning, and the training they had received.

### Physical therapy university students

Participants were 4 (3 females, 1 male; age of 22 years) last year physical therapy students that took part in the S-L program. After the qualitative analysis, three categories emerged, reflecting the experiences of physical therapy in the S-L program: Motivations for the project, Learnings, and Positive and negative aspects of the service. Table 4 shows a display of qualitative themes and subthemes.

1. *Motivations for the project.*

Physical therapy students reflected on the reasons that motivated them to choose the S-L project. Three subcategories emerged.

- Fieldwork with real patients:

Some of the participants found the idea of working with real patients very interesting instead of just writing a theoretical memory.

“I wanted to choose a research project to do something different for my final degree project. As I had already done clinical cases during my internship program, I wanted to do something interesting that brought me more than I had already done during the 4 previous years” P.2.

- Work with children:

One of the motivations that physical therapy students argued for choosing this project was the population to work with, they looked forward to teaching children.

“I was motivated by the procedure of the project, conducting the musculoskeletal education in schools. I consider that I like primary education children, and I can easily adapt to them” P.4.

- Opportunity to solve the needs of a population:

Physical therapy students highlighted the value of offering their service in a population where participants had no prior knowledge of postural hygiene.

“The need to raise the population awareness, in particular children, about the importance of healthy habits to prevent musculoskeletal problems in the future” P.3.

2. *Learnings.*

They described which competencies they acquired through their service, not only professionally but also personally. Three subcategories emerged.

- Increasing professional skills:

Physical therapy students described how they increased their knowledge in aspects such as research methodology and general and specific professional skills.

“We have never prepared before a talk with an audience without any previous knowledge on medicine or physical therapy. Besides, we have learned to adapt to the different ages of the children, to the unexpected situations, to the different questions for explaining and better sharing our knowledge with them” P.1.

- Children-friendly approach:

Participants highlighted the process they followed to adapt the content to the young population and how they learned to interact with children.

“When providing this service to the child population, what I have learned the most is how to deal with groups of children for the proper development of the activity” P.3.

- Future application of the knowledge:

They explained the impact of the service on themselves and on their future as physical therapists and researchers.

“I will have more conscience about care of my own body. And above all, I consider that I have learned to explain knowledge to the children, I like them a lot and I’m sure that this will not be the last time that I do it” P.4.

3. *Positive and negative aspects of the service.*

Physical therapy students experienced not only positive but also challenging moments during the service.

- Facing difficulties:

Participants sometimes experienced difficult situations that they had to deal with, feeling stress or fear, but they found strategies to resolve these situations successfully.

**Table 4:** Summary of qualitative data analysis.

Themes	Subthemes
Theme 1: Motivations for the project	Fieldwork with real patients
	Work with children
	Opportunity to solve the needs of a population
Theme 2: Learnings	Increasing professional skills
	Children-friendly approach
	Future application of the knowledge
Theme 3: Positive and negative aspects of the service	Facing difficulties
	Aspects to be improved
	Usefulness of the service
	Satisfaction with the service

“At the beginning, I was very afraid, then I felt more confident, and I enjoyed it a lot” P.2.

- Aspects to be improved:

They explained how the service could be improved in the future, and what strategies schools could implement to increase awareness of pupils ‘positional hygiene.

“…with a better organisation of the time schedules and places of presentation such, for example, try to avoid the sports hall because there is a lot of echoing and children cannot listen well” P.1.

- Usefulness of the service:

Physical therapy students explained the benefits that the school children gain from the service provided.

“…it was very useful for us, for children’s teachers and, above all, for children…at the end of the presentation’s children were more conscient of their bodies and of their position when being sitting in class, they also learnt to carry properly the weight of their backpack. They were curious and got interested in practicing self-massage at home to use it at their homes” P. 3.

- Satisfaction with the service:

Physical therapy students highlighted the positive aspects they perceived as students and the benefits for their future professional careers.

“I loved it, living this experience was so interesting for me and it will surely bring to me a lot as a physical therapist, either working with children or with adults. It has been very important for me to teach pupils self-care and body awareness.” P. 1.

## Discussion

This study examines the learning processes, experiences, and satisfaction of both school students and teachers within the school community, following their participation in a MSKHPP, which was carried out during two academic courses, reaching a total of 1,051 children and adolescents. The program was designed according to S-L principles and implemented by fourth-year physical therapy students, whose experiences were qualitatively explored.

For decades, peer education (46) and university learning through S-L projects (47) have been widely studied. This study uniquely combines both models by promoting reciprocal learning between two educational levels—school and university—while integrating teaching-based learning. Additionally, a third group, the schoolteachers, acted as learners and mediators, contributing to and benefiting from the teaching process. Together, these participants engaged in a collaborative effort that led to meaningful mutual learning.

The present findings provide preliminary evidence of the effectiveness of health promotion programs in the school community, particularly those that emphasize body awareness, prevention, healthy habits and self-management of MSKD. Other studies addressed health education within the school community and focused primarily on back posture (48), increasing physical activity (49, 50), improving nutritional habits (49), or reducing obesity (51, 52). However, this study takes a different approach by using a multimodal and targeted strategy specifically at the musculoskeletal system level. The development of both theoretical and practical interventions has been shown to be effective in facilitating the acquisition of new knowledge (48), and our results contribute to the need of growing the evidence in this area.

Significant improvements were observed in both schoolchildren’s and teachers’ knowledge related to the locomotor system, physical therapy, and the prevention of MSKD. A better understanding of musculoskeletal health is crucial for empowering individuals to make informed health decisions, a concept increasingly recognized as health literacy (HL) (53). According to Sorensen et al. (54), HL encompasses not only the necessary knowledge but also the motivation and skills required to maintain or improve one’s quality of life. The findings of the present study are consistent with the review and meta-analysis by García-Moreno et al. (55), who reported that physical therapy positively influences behavior and knowledge related to back care and the prevention of non-specific low back pain in children and adolescents. Regarding self-body awareness and posture, the results of this study are consistent with those of Minghelli et al. (56, 57), where an improvement in ergonomic knowledge was achieved through a back postural education program. However, these results were obtained through theoretical and practical tests, while the present results were gathered using a questionnaire.

This study also demonstrated the benefits of guided practical activities such as flexibility, strengthening, breathing, and self-treatment, which is in line with the work of Cardon et al. (58), suggesting that prevention of MSKD pain is more effective when active strategies are employed, compared to programs that focus solely on postural hygiene. Notably, few school-based interventions prioritize learning for the prevention of MSKD or offer opportunities for school students to practice self-treatment techniques as preventive measures, making our approach particularly valuable. Similarly, Mirskaya et al. (59) developed a specialized model for the prevention and correction of MSKD in school students, achieving a 50% reduction in prevalence among students in several Moscow schools. This model enhances the early detection of musculoskeletal pathologies in school-age children and supports ongoing prevention efforts within educational settings (60).

In addition, both school students and teachers expressed an increased interest in education on the prevention of MSKD. It is important to highlight that health promotion goes beyond simply encouraging children and adolescents to eat healthily and be physically active; it is embedded in a broader approach that relies on active engagement with the surrounding community (61, 62). The increased interest observed in both groups may be attributed to the engagement fostered by our study, which provided quick and available feedback and facilitated reflective practice through a combination of theoretical and practical teaching (63). This has also been accomplished through close collaboration between all school stakeholders—including teachers, school students, and community members—to create a transformative educational process, as highlighted in the study by David and Cooke (64).

School students reported a high level of satisfaction with the MSHPP, citing their increased knowledge of the human body, understanding of musculoskeletal disorders, and insights into prevention strategies as key contributors. For teachers, active participation in such programs was highlighted as the most significant aspect, supporting that continuous professional development and training are essential for them to effectively promote musculoskeletal health. These findings align with those of Rodrigo-Sanjoaquín et al., (62) who conducted a qualitative study on the challenges and opportunities in the implementation of Aragon’s Health-Promoting School network. Their research underscores the central role of teachers in school health promotion, pointing to their ideal position to design and implement tailored health programs (65). Similarly, the study by Hung et al. (66) emphasize the importance of providing teachers with education and training on health-related topics. Through the training, the teachers´ confidence in their ability to continue health education in the courses could be enhanced, optimizing the impact of the program in the long term.

As can be seen, the challenge of implementing training within school-based programs is engaging all relevant stakeholders. Durl et al. (67) demonstrated that integrating insights from both students and teachers enhances program effectiveness, particularly when a diverse group of stakeholders—including end users—is actively involved in key stages of co-creation (design, implementation, and evaluation). Our study aligns with the findings of this author, demonstrating a collaborative effort that fostered meaningful mutual learning. A scoping review identified several practical challenges can limit the effectiveness and reach of school-based interventions, including teacher training, academic priorities, limited resources, and staff workload. Additionally, it highlighted that evaluating these interventions is rarely included as a formal metric. However, systematically assessing intervention outcomes is crucial to understanding which interventions benefit specific populations. Incorporating rigorous evaluation informs decisions on intervention dissemination and future implementation strategies and ensures continuous assessment and refinement of interventions into practice (68).

This project was developed by physical therapy university students as part of their final degree project through a S-L methodology (69). Preparing the sessions, adapting to a school audience, improvising during the workshops, and guiding practical exercises required to deeply study the material in order to teach it effectively and address any questions that arose. This process fostered significant learning, both in knowledge and skills, through the mechanism of implicit learning. Learning by teaching, a method introduced in the 1980s and refined by Grzega and Schöner (70), enables students to achieve autonomous and deep learning. Participating students reflected on their learning experience, noting that it enhanced their skills as future health professionals. For physical therapists, effective communication with patients is essential, both during anamnesis and in establishing a strong therapeutic alliance (71, 72). Communicating health information to school children improved their communication skills as they had to adapt their message to an audience with different language skills and levels of knowledge. A similar study conducted in Mexico with engineering university students echoes these findings, emphasizing the benefits of learning through teaching (73). One student underscored the importance of going beyond written and purely cognitive learning to engage in real-world contexts. As noted in their reflections, this approach heightened their motivation toward such educational systems. Providing S-L experiences that include opportunities for reflection throughout education enhances university students’ understanding of social responsibility (74). Moreover, as future practitioners implementing S-L initiatives, physical therapy students must be prepared to operate within a rapidly evolving healthcare environment, which now demands greater community accountability.

Despite the promising results, policy and practical implications remain underexplored. School-based health promotion interventions, particularly those focused on musculoskeletal health, have the potential to be integrated into public health policies as a cost-effective strategy to reduce long-term MSKD-related burden. The inclusion of structured musculoskeletal health curricula in schools could help bring about sustainable behavioral changes that persist into adulthood and contribute to the prevention of chronic conditions such as low back pain (67). Future research should explore the feasibility of implementing these programs on a larger scale, ensuring collaboration between educational and healthcare public systems.

This study, designed to implement and assess a MSKHPP in schools, demonstrated its innovative nature by specifically targeting musculoskeletal disorders. A multimodal approach was developed, combining theoretical and practical methodologies to teach prevention and self-management of MSKD. However, it has certain limitations. First, the lack of family involvement, which could have enhanced the program’s effectiveness by reinforcing the learned behaviors at home. Evidence suggests that parental engagement plays a crucial role in maintaining health-promoting behaviors in children and adolescents (75). Additionally, the long-term impact of the intervention was not assessed, and further studies should incorporate objective testing methods and follow-up assessments to determine retention of knowledge and behavioral changes over time. Nevertheless, the extension of this program to two academic courses allowed it to reach a large population, supporting not only its usefulness in terms of MSKD prevention but also the sustainability of these interventions when conducted through S-L.

## Conclusion

In conclusion, MSKHPP delivered by physical therapists in the school community, can contribute to learning useful resources for musculoskeletal pain prevention and self-management. In addition, health promotion initiatives developed using S-L could be a cost-free alternative to the current gaps in public health policies, while qualitatively improving the education of future healthcare professionals.

## Data Availability

The raw data supporting the conclusions of this article will be made available by the authors, without undue reservation.
